# Genomic signatures characterize leukocyte infiltration in myositis muscles

**DOI:** 10.1186/1755-8794-5-53

**Published:** 2012-11-21

**Authors:** Wei Zhu, Katie Streicher, Nan Shen, Brandon W Higgs, Chris Morehouse, Lydia Greenlees, Anthony A Amato, Koustubh Ranade, Laura Richman, David Fiorentino, Bahija Jallal, Steven A Greenberg, Yihong Yao

**Affiliations:** 1 Translational Sciences, MedImmune, LLC, One MedImmune Way, Gaithersburg, MD, 20878, USA; 2 Shanghai Renji Hospital, Shanghai Jiaotong University School of Medicine, Shanghai, China; 3 Department of Dermatology, Stanford University School of Medicine, Palo Alto, CA, USA; 4 Department of Neurology, Brigham and Women’s Hospital Harvard Medical School, Boston, MA, USA; 5 Children’s Hospital Informatics Program, Harvard Medical School, Boston, MA, USA

**Keywords:** Myositis, Genomics, Leukocyte infiltration, Type 1 interferon, miR-146a

## Abstract

**Background:**

Leukocyte infiltration plays an important role in the pathogenesis and progression of myositis, and is highly associated with disease severity. Currently, there is a lack of: efficacious therapies for myositis; understanding of the molecular features important for disease pathogenesis; and potential molecular biomarkers for characterizing inflammatory myopathies to aid in clinical development.

**Methods:**

In this study, we developed a simple model and predicted that 1) leukocyte-specific transcripts (including both protein-coding transcripts and microRNAs) should be coherently overexpressed in myositis muscle and 2) the level of over-expression of these transcripts should be correlated with leukocyte infiltration. We applied this model to assess immune cell infiltration in myositis by examining mRNA and microRNA (miRNA) expression profiles in muscle biopsies from 31 myositis patients and 5 normal controls.

**Results:**

Several gene signatures, including a leukocyte index, type 1 interferon (IFN), MHC class I, and immunoglobulin signature, were developed to characterize myositis patients at the molecular level. The leukocyte index, consisting of genes predominantly associated with immune function, displayed strong concordance with pathological assessment of immune cell infiltration. This leukocyte index was subsequently utilized to differentiate transcriptional changes due to leukocyte infiltration from other alterations in myositis muscle. Results from this differentiation revealed biologically relevant differences in the relationship between the type 1 IFN pathway, miR-146a, and leukocyte infiltration within various myositis subtypes.

**Conclusions:**

Results indicate that a likely interaction between miR-146a expression and the type 1 IFN pathway is confounded by the level of leukocyte infiltration into muscle tissue. Although the role of miR-146a in myositis remains uncertain, our results highlight the potential benefit of deconvoluting the source of transcriptional changes in myositis muscle or other heterogeneous tissue samples. Taken together, the leukocyte index and other gene signatures developed in this study may be potential molecular biomarkers to help to further characterize inflammatory myopathies and aid in clinical development. These hypotheses need to be confirmed in separate and sufficiently powered clinical trials.

## Background

Myositis is characterized clinically by skeletal muscle weakness and histopathologically by the presence of inflammatory cells in muscle tissue. There are several major subclasses of myositis, including dermatomyositis (DM), polymyositis (PM), inclusion body myositis (IBM), and immune mediated necrotizing myopathy (NM). The leukocyte infiltration present in myositis muscle is believed to contribute to disease pathogenesis [1-5]. The types of immune cells present in myositis muscle were originally identified in the 1980s as predominantly CD4+ T cells and B cells in DM, and CD4+ and CD8+ T cells in IBM [1,6-8], with more recent identification of plasmacytoid dendritic cells in DM [9], myeloid dendritic cells in PM and IBM [3], and plasma cells in all three disorders [2]. Unlike DM, PM or IBM, NM is characterized by myofiber necrosis associated with macrophages and minimal T cell infiltration or MHC Class I expression [10]. Given the differences in clinical manifestations between these subtypes of myositis and the lack of optimal efficacious therapies for these diseases, understanding the molecular characteristics underlying their subtypes may facilitate the development of novel therapeutics that could benefit patients with myositis.

Technologies such as whole genome microarray have advanced our understanding of the disease pathogenesis of myositis [11-13]. A large number of type 1 interferon-stimulated genes (ISGs) were identified to be strongly overexpressed in DM muscle and this molecular signature has been further confirmed by recent studies [4,14]. The activation of type 1 interferon (IFN) signaling has been observed in many autoimmune diseases [15,16], such as systemic lupus erythematosus (SLE) [17-19], systemic sclerosis [20], rheumatoid arthritis [21], and psoriasis [22]. A type 1 IFN signature is not only present in DM muscle but also expressed in DM skin [23], as well as peripheral blood of DM and PM, reflecting disease activity [24-26]. Based on the accumulating evidence from recent microarray studies and other complementary experiments, disease models have been proposed to emphasize the central role of type 1 IFN pathway activation in the pathogenesis of DM, suggesting that blockade of type 1 IFN might provide clinical benefit to DM patients [27].

In addition to previous studies focused on altered mRNA expression in myositis, the roles of microRNAs (miRNAs) in regulating immune responses, muscle development, and regeneration are also emerging [28-31]. miRNAs including miR-146a, miR-155, and miR-101 have been shown to be aberrantly expressed in rheumatic diseases [32-35] and miR-1, miR-133a/b, and miR-206 have been identified as muscle-specific miRNAs critical for muscle development and function [28-31]. Though the role of miRNAs in the pathogenesis of myositis has yet to be evaluated extensively, it is worth noting that interactions between miRNAs and type 1 IFN have been identified [36]. Specifically, miR-146a suppresses the innate immune response not only via the TLR-mediated NFκB pathway [37], but also negatively regulates the type 1 IFN pathway in SLE by targeting STAT1 and IFN regulatory factor 5 (IRF5) [38].

Despite the aforementioned studies, there still lacks a clear understanding of the disease pathogenesis that underlies myositis. Meanwhile, few molecular biomarkers have been identified to aid in stratifying myositis patients or objectively quantifying the leukocyte abundance in inflammatory muscle and the corresponding muscle fiber damage. To address the unmet needs in these areas, we performed both genome-wide mRNA and miRNA expression profiling in muscle biopsies from myositis patients and normal controls. Our studies revealed gene expression signatures specific for myositis and distinct for each subclass of myositis, as well as multiple pairs of mRNA:miRNA displaying anti-correlation expression patterns in line with predicted relationships. Additionally, expression data from this study indicated that miR-146a displayed a positive correlation with the type 1 IFN signature rather than the expected negative correlation in myositis muscle. We postulated that such positive correlation could be driven by infiltrated leukocytes; therefore, we developed an invasion model to account for transcriptional changes due to leukocyte infiltration. Further analyses with this invasion model indicated that the source of ISG expression may differ between subtypes of myositis, such that in PM and IBM, ISG expression is associated with infiltrated leukocytes, whereas in DM, non-leukocyte cells (e.g., muscle cells) might contribute significantly to ISG expression. Collectively, our results revealed multiple gene expression signatures that can potentially advance our understanding of the pathologic characteristics of myositis and provide utility as molecular biomarkers for identifying the right therapeutics for myositis patients.

## Results

### Altered mRNA and miRNA expression reveals upregulation of immune pathways and downregulation of muscle contraction pathways in myositis

A total of 837 probesets that represent 606 unique genes were differentially expressed by two-group *t*-tests in muscle specimens of myositis patients compared with non-neuromuscular disease patients (Additional file 1: Table S1). Most differentially expressed transcripts (88%, 740 of 837) were over-expressed, with only 97 (12%) down-regulated. Functional analysis using IPA (Ingenuity Pathway Analysis, http://www.ingenuity.com/) and gene set enrichment showed that the most upregulated genes are involved in the immune and inflammatory responses, including a large number of ISGs, MHC class I/II proteins, and genes associated with natural killer cell mediated cytotoxicity. In contrast, muscle contraction and calcium signaling pathway-related genes are enriched in the down-regulated transcripts (*p*-value < 0.001 and *p*-value = 0.001, respectively). In addition to the identification of differentially expressed transcripts in myositis compared to normal controls, we also identified subclass-specific differentially expressed mRNAs by comparing each myositis subtype to the normal controls (Additional file 2: Supplementary Text; Additional file 2: Figure S2; Additional file 3: Table S2).

A similar statistical analysis to determine differentially expressed mRNAs was applied to miRNA expression data to identify differentially expressed miRNAs. Of 69 differentially expressed miRNAs identified when comparing myositis muscle with normal controls, only five were over-expressed (miR-146a/b, miR-155, miR-21 and miR-432). The remaining 64 miRNAs were down-regulated, including miR-133a/b, miR-1, and miR-29c (Additional file 4: Table S3). Notably, both miR-146a and miR-155 are predominantly expressed in leukocytes and also play important roles in regulating immune responses [32-35].

**Figure 1:**
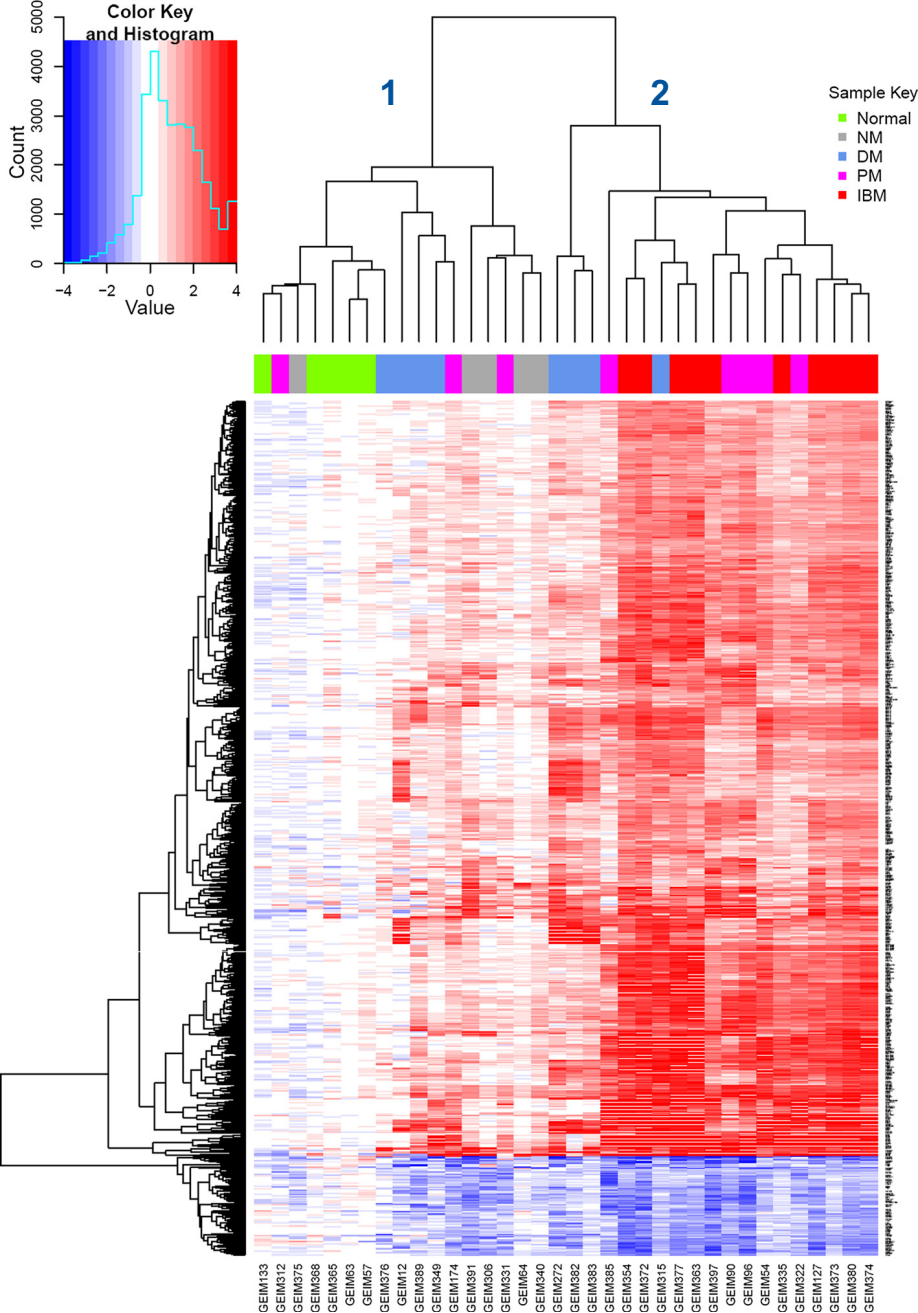
**Heatmap of 837 differentially expressed transcripts.** Transcript expression fold change is represented by the color scale increasing from blue to white to red; the color key is shown in the top left corner. The 36 subjects are shown horizontally and represented by the color bar on the top of the heatmap. The color that corresponds to each disease type is displayed in the upper right corner. At the first bifurcation of the horizontal dendrogram, the subjects are classified into 2 groups from left to right: group 1 and group 2.

Unsupervised hierarchical clustering of the 837 differentially regulated mRNA transcripts revealed two main transcriptionally-defined subcategories, with all normal and NM samples clustered within one branch (Figure 1, branch 1), and all IBM samples clustered within the other branch (Figure 1, branch 2). DM and PM samples are divided into both branches. Although unavailable for the patients within this study, additional information such as myositis-specific antibodies and other serology data could have contributed to a more optimal phenotyping of the myositis specimens. The lack of these data may explain some of the heterogeneity observed within the groups defined strictly by the diagnostic criteria NM, DM, PM, and IBM. Nevertheless, our results suggest that specific molecular expression patterns, in addition to others previously defined for myositis, might provide insight into classifying and understanding the pathophysiology of these different subtypes of myositis.

### Consensus clustering reveals five gene signatures that characterize myositis

In addition to identifying molecular expression patterns specific for myositis as a whole and distinct for its individual subtypes, we also applied and merged multiple clustering algorithms to identify robust clusters of genes that may provide unique insight into the molecular components of myositis (see Methods for the details). To rule out uninformative genes, we selected the top 200 genes with the most varied expression from the 606 differentially expressed genes, among which, only three were down-regulated. Therefore, we focused on the remaining 197 up-regulated genes and clustering produced five clusters with robust gene members (i.e., cluster A-E; Additional file 2: Figure S2C and S3; also see Methods).

**Figure 2:**
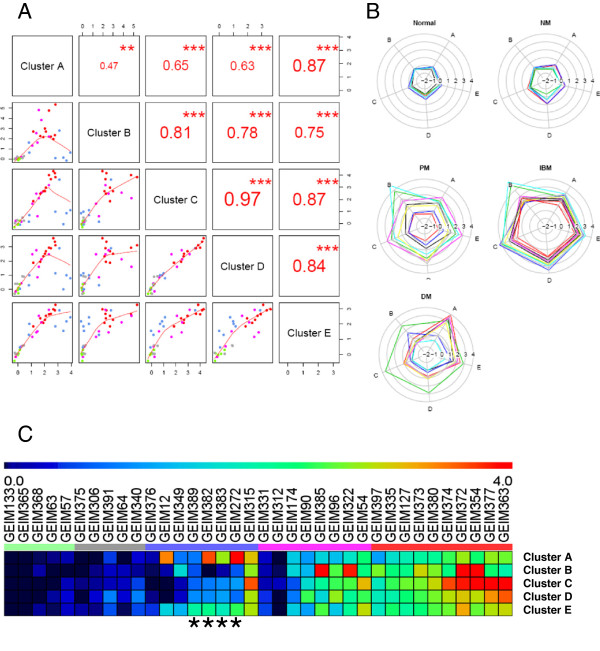
**Gene signatures derived from clusters A-E from the consensus cluster. **(**A**) Pairwise scatter plots of the five gene signatures. The scatter plots of the signatures of the clusters A-E are displayed in the lower panels, where data points are colored by the sample type (same as the Figure 1) and red lines represent the lowess (Locally Weighted Scatterplot Smoothing) lines. The corresponding spearman correlation coefficients are displayed in the upper panels, with significance levels indicated by the stars (*** p < 0.001, ** p < 0.01, * p < 0.05). (**B**) Subclasses of myositis characterized by the five gene signatures are shown in the star plots. Each subject is represented with a specific pentagon in the star plot and the vertices of one pentagon along the spokes (A-E) represent enrichment of the signature scores from clusters A-E. Colors of pentagons are randomly assigned to distinguish subjects and star plots are grouped by the myositis subclasses. (**C**) Subjects can be further characterized by the five gene signatures using a heatmap. The signature scores are represented by the rainbow color in the heatmap, ranging between 0 (colored in blue) and 4 (colored in red); the color key for each sample’s disease type is displayed horizontally between the sample identifiers and the heatmap, where, (from left to right), green denotes normal, gray for NM, blue for DM, magenta for PM and red for IBM. The four DM patients with perifascicular atrophy (PFA) are marked by the star symbol.

Subsequent functional analyses of the five clusters revealed that clusters A and B were enriched with type 1 ISGs and immunoglobulins, respectively; clusters C and D had very similar profiles (Figure 2A) and were enriched with immune response-related genes (Additional file 5: Table S4); and cluster E was dominated by MHC class I genes. These observations led us to define five gene signatures enriched in myositis muscle tissue: type 1 IFN signature (from cluster A), immunoglobulin signature (from cluster B), leukocyte I and II indices (derived from clusters C and D, respectively) and MHC class I signature (from cluster E). The similarity in cluster results shared by the different clustering methods confirmed the scientific rigor of this analysis (Additional file 5: Table S4). We then proceeded to analyze disease heterogeneity within each myositis subclass by utilizing these five gene signatures (Figure 2B-C). In strong agreement with results shown in Figure 1, NM displayed homogeneous gene signature profiles similar to normal muscle, with a slightly elevated leukocyte II signature observed (see Figure 2B-C). IBM exhibited clear over-expression of all five gene signatures. Gene signatures in PM and DM varied from normal-like signatures to patterns similar to IBM. Most myositis patients showed coordinate overexpression across all modules (shown as an equilateral right pentagon in the radial plot; Figure 2B), but DM showed some heterogeneity of relative overexpression of the type 1 IFN module (i.e., cluster A).

Both leukocyte index I and II are comprised of over-expressed genes related to immune function (Additional file 5: Table S4); displayed differences in magnitude between myositis patients; and are highly correlated with each other (*Spearman* rank test; *r* = 0.97, *p* < 0.001; see Figure 2A). Therefore, we selected leukocyte index I (generated from cluster C) as the leukocyte infiltration index for subsequent analyses examining altered gene expression due to increased immune cell infiltration into muscle.

### A leukocyte index distinguishes altered expression of transcripts due to immune cell infiltration and correlates with histopathology results

In myositis, leukocyte infiltration and/or clonal expansion results in a marked increase in the abundance of leukocytes in muscle. Consequently, the interpretation of gene expression studies in myositis tissue biopsies can be greatly influenced by immune cell infiltration. In a principal components analysis of the 837 differentially expressed transcripts in this study, we found that the first principal component (PC1) accounted for 75.6% of the variance in the data. PC1 had a significant linear association with our proposed leukocyte index (*R*^*2*^=0.92, *p*<0.001), suggesting that the majority of the overall expression variation was due to the change of leukocyte cell numbers and could be accounted for by this index. This computational representation of leukocyte infiltration could eliminate the transcriptional variation generated solely from the increased immune cell proportion in muscle samples and allow gene expression changes to be attributed to the appropriate source.

**Figure 3:**
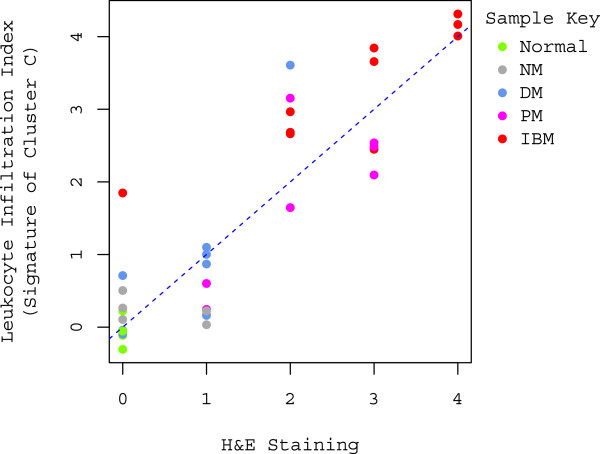
**Correlation between leukocyte infiltration index and H&E staining. **A significant linear correlation is displayed in the scatter plot between the leukocyte infiltration index and the degree of inflammation assessed by H&E staining (0=no inflammation, 1=sparse inflammation, 2=mild inflammation, 3=moderate inflammation, and 4=severe inflammation). Data points are colored by the sample type; color key is displayed in the upper right corner of the plot. The regression line is denoted by the blue dotted line (intercept=0.07, slope=1.00; *R*^*2*^=0.79, *p*<0.001).

The ability of the leukocyte index to quantify the extent of leukocyte infiltration is indirectly supported by the significant enrichment of immune response-related transcripts among the differentially expressed transcripts (hypergeometric test, *p*< 0.001), as well as the robust clustering of these genes. Additionally, there is a significant linear correlation between the leukocyte index and immune cell infiltration identified and scored by hematoxylin and eosin (H&E) staining (*R*^*2*^=0.79, *p*<0.001; Figure 3). This provides direct evidence to support the leukocyte infiltration index described here by confirming that the index accurately reflects leukocyte abundance in muscle.

In addition to this ability of the leukocyte index to correctly reflect the abundance of immune cells in muscle tissue, it may also inversely correlate with the expression of muscle-specific mRNA or miRNA due to either a decreased proportion of muscle cells in the sample or muscle damage. For instance, TTN is a structural protein abundant in myofibers whose deficiency was found to be associated with DM perifascicular atrophy (DM-PFA) [14]; miR-1 is a muscle specific miRNA [39] that has an important role in muscle development [28-31]. Both TTN and miR-1 exhibited a marked anti-correlation with the leukocyte index (Spearman rank test; *r* = −0.70, *p*<0.001 and *r* = −0.64, *p*<0.001, respectively), providing additional evidence to support the validity of our invasion model and the derived leukocyte index.

### Multiple mRNAs dysregulated in myositis muscle are not due to leukocyte infiltration

The development of the leukocyte infiltration index revealed that most of the over-expressed transcripts in myositis muscle compared to normal muscle specimens were not truly up-regulated due to transcript over-expression, but rather these expression changes were actually due to leukocyte cells infiltrating the muscle tissue. Accordingly, we adjusted the expression data matrix using the infiltration index to identify over-expressed transcripts unaccounted for by leukocyte infiltration. Consequently, only 41 (5.5%) out of 740 over-expressed mRNAs remained over-expressed after adjustment with the infiltration index (Additional file 6: Table S5). These miRNAs included many immunoglobulins (IGK, IGH, IGL), chemokines (CCL8, CCL11, CCL18), myosin complex genes, and cytoskeletal genes (POSTN, TNNT2 and MYH8). The dramatic reduction of over-expressed transcripts after adjusting for leukocyte infiltration confirmed that most of the over-expression is driven by increased leukocyte abundance in myositis muscle.

Subclass-specific transcript analysis following adjustment for leukocyte infiltration (Additional file 7: Table S6) revealed that immunoglobulin proteins and some chemokines were over-expressed in both IBM and PM, whereas ISGs were heavily over-expressed in DM muscle. Overall, our results suggested that myofibril and chemokine signatures exist in DM, PM and IBM, and likely resulted from changes in gene expression in the muscle cells themselves rather than alterations due to leukocyte infiltration. Understanding the contribution of these pathways altered in muscle tissue may provide keen insight into the pathogenesis of specific subclasses of myositis.

### The leukocyte index and the type 1 IFN signature in myositis muscle

**Figure 4:**
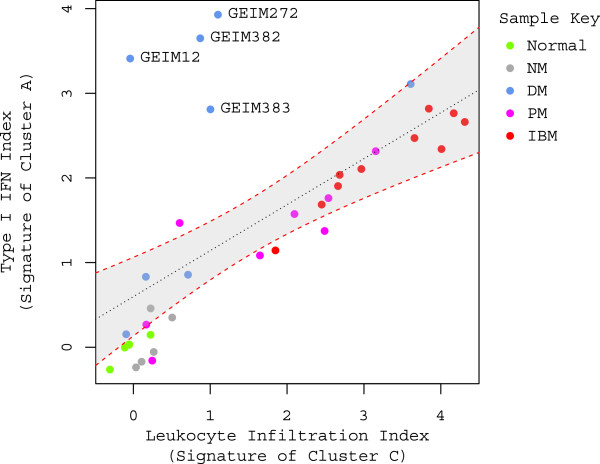
**Correlation between type 1 IFN signature score and leukocyte infiltration index in myositis. **A significant linear correlation is displayed in the scatter plot between type 1 IFN index and leukocyte infiltration index. Data points are colored by the sample type; color key is displayed in the upper right corner of the plot. The regression line (*R*^*2*^=0.41, *p*<0.001) is denoted by the black dotted line and 95% confidence interval is outlined by the red dotted line and gray shaded background. The subject identifiers of the four outliers are labeled. When excluding the four outliers, the *R*-squared value in the regression analysis escalates from 0.41 to 0.91.

After re-assessing gene expression changes to account for leukocyte infiltration, we observed that ISGs remained highly over-expressed in muscle biopsies particularly from DM patients. Accordingly, we investigated the relationship between over-expression of ISGs and the leukocyte index in more depth. Strikingly, the type 1 IFN signature showed a significant linear correlation with the leukocyte index with exception of four outliers (i.e., the DM subjects GEIM 12, 272, 382 and 383; see Figure 4). The linear correlation suggests that leukocyte infiltration might have a more pronounced effect on ISG over-expression in IBM, PM, and DM without perifascicular atrophy (PFA; see Table 1); whereas in a subset of DM patients with marked ISG over-expression, especially those with PFA, other cell types (e.g., muscle fiber cells) might contribute to type 1 IFN pathway activation.

**Table 1:** Patient information

***Subject ID***	***Age***	***Gender***	Disease type*	***Muscle***	***Disease duration (months)***	***Treatments***
GEIM12	74	F	DM	Biceps	1	None
GEIM54	62	M	PM	Quadriceps	24	None
GEIM57	38	F	Normal	Biceps	N/A	N/A
GEIM63	37	F	Normal	Biceps	N/A	N/A
GEIM64	77	M	NM	Biceps	6	Pred
GEIM90	90	M	PM	Deltoid	3	None
GEIM96	86	F	PM	Biceps	3	None
GEIM127	68	F	IBM	Biceps	36	Azathioprine
GEIM133	60	F	Normal	Quadriceps	N/A	N/A
GEIM174	36	F	PM	Biceps	3	None
GEIM272	25	F	DM (with PFA)	Biceps	3	Pred x 4 days
GEIM306	64	F	NM	Biceps	17	Pred
GEIM312	76	F	PM	Biceps	96	None
GEIM315	39	F	DM	Biceps	3	None
GEIM322	58	F	PM	Biceps	180	None
GEIM331	87	F	PM	Deltoid	36	Pred
GEIM335	71	F	IBM	Quadriceps	24	None
GEIM340	83	F	NM	Biceps	66	Pred
GEIM349	28	F	DM	Biceps	18	Pred, Azathioprine
GEIM354	76	M	IBM	Biceps	60	None
GEIM363	79	F	IBM	Biceps	36	None
GEIM365	41	M	Normal	Biceps	N/A	N/A
GEIM368	61	F	Normal	Quadriceps	N/A	N/A
GEIM372	61	M	IBM	Quadriceps	84	Dexamethasone taper
GEIM373	80	F	IBM	Quadriceps	90	None
GEIM374	47	F	IBM	Quadriceps	54	None
GEIM375	50	F	NM	Quadriceps	6	None
GEIM376	37	M	DM	Deltoid	4	None
GEIM377	50	M	IBM	Biceps	24	None
GEIM380	61	M	IBM	Quadriceps	24	None
GEIM382	30	F	DM (with PFA)	Deltoid	1	None
GEIM383	44	F	DM (with PFA)	Deltoid	2	Pred x 1 month
GEIM385	61	F	PM	Biceps	60	Etancercept
GEIM389	40	M	DM (with PFA)	Biceps	5	N/A
GEIM391	71	M	NM	Quadriceps	7	None
GEIM397	77	M	IBM	Biceps	84	None

* DM, dermatomyosits; PM, polymyositis; IBM, inclusion body myositis; PFA, perifascicular atrophy.

**Figure 5:**
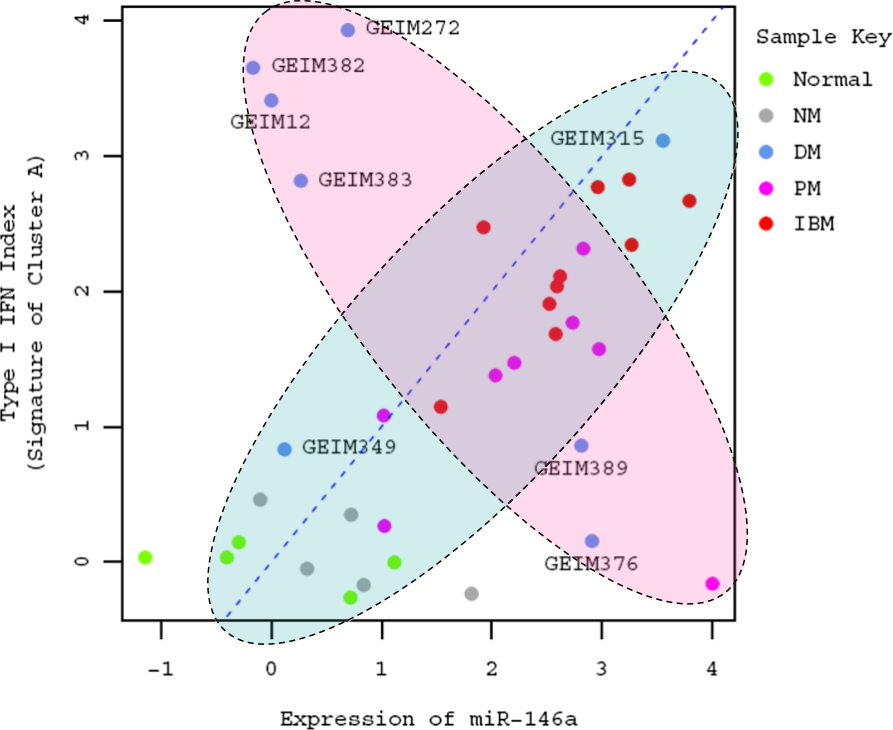
**Intricate association between the expression of miR-146a and type 1 IFN signature score in a subclass of myositis. **Data points are colored by the sample type in this scatter plot; color key is displayed in the upper right corner of the plot. The identity reference line *y*=*x* is denoted by the blue dotted line. Type 1 IFN signature displays a positive pattern of association in most subjects including the normal control, NM, PM, IBM and some DM, as highlighted in the light blue shaded oval. On the other hand, the type 1 IFN signature displays a negative pattern of association with the expression level of miR-146a in most DM (highlighted in the pink shaded oval). The subject identifiers of the eight DM subjects are all labeled in this plot.

Observations that a correlation between ISG over-expression and leukocyte infiltration varied between myositis subtypes prompted us to also investigate the correlation of miR-146a with the leukocyte index as this miRNA has been previously reported to negatively regulate the type 1 IFN pathway in SLE [38]. Our results indicated that both miR-146a and the type 1 IFN signature display an overall positive correlation with the leukocyte index (*Spearman* rank test; *r* = 0.64, *p* < 0.001; *r* = 0.72, *p* < 0.001, respectively; also see Figure 4), and therefore they are likely to be positively correlated with each other as well. Upon further evaluation, this positive pattern of association was evident primarily in IBM and PM (Figure 5), where leukocyte infiltration may be responsible for the observed alterations in miR-146a expression. In contrast, 6 out of 8 DM samples displayed a negative pattern of association between the expression level of miR-146a and the type 1 IFN signature, similar to what has been shown previously in SLE [38].

**Figure 6:**
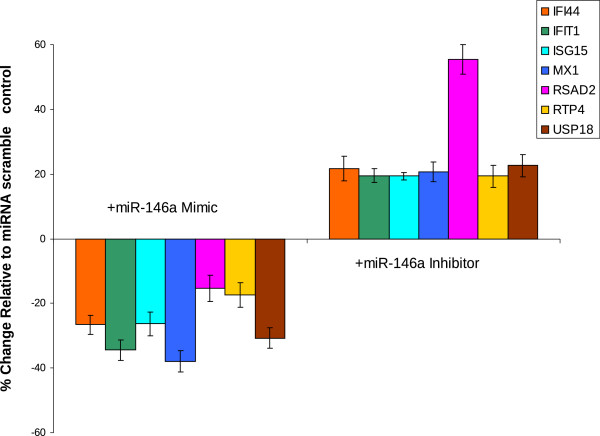
**miR-146a alters the expression of type 1 IFN inducible genes in C2C12 muscle cells. **C2C12 cells were transfected with miR-146a mimics and miR-146a inhibitors, as well as the appropriate scrambled controls. 24 hours after transfection, cells were stimulated with mouse IFN-alpha for 4 hours before RNA was isolated and profiled for alterations in ISGs. Regulation of the IFN pathway was demonstrated by increased ISG transcript levels following inhibition of miR-146a, as well as decreased ISG transcript levels following addition of miR-146a. Values are shown as mean ± SEM and represent results from 3 independent experiments.

To examine the possibility that miR-146a could negatively regulate the type 1 IFN signature in muscle tissue, we utilized an *in vitro* muscle cell model. The level of miR-146a expression was altered in C2C12 muscle cells by transfecting either miR-146a mimics or miR-146a inhibitors into this cell line prior to stimulation with type 1 IFN. Measuring the resulting expression levels of ISGs illustrated that miR-146a is able to negatively regulate the type 1 IFN pathway in muscle cells (Figure 6), as demonstrated by the increased ISG expression following inhibition of miR-146a and the decreased ISG expression following increased miR-146a levels. These trends and degree of effect on ISGs by miR-146a are in agreement with what has been reported previously in SLE [38], and suggest a possible role for miR-146a in regulating the type 1 IFN pathway in muscle, although this remains to be verified by *in vivo* studies.

## Discussion

Most biological tissue specimens are heterogeneous, often consisting of multiple cell types. This heterogeneity is evident in myositis muscle biopsies and confounds transcriptional analyses, as the source of an observed expression change is unclear. We developed a leukocyte index to differentiate the transcriptional alterations due to an increased proportion of inflammatory cells from other changes at the expression level. The positive correlation of this computational leukocyte index with immune cell infiltration scored from H&E staining and the expression of lymphocyte-specific mRNAs/miRNAs validated our invasion model and further confirmed the use of this index as a surrogate for leukocyte abundance in muscle. The ability to distinguish between muscle-derived transcripts and genes over-expressed due to leukocyte infiltration may improve the interpretation of gene expression results and increase existing knowledge of molecular pathways critical for myositis.

In addition to the leukocyte index, we have also identified several other gene signatures (each from a consensus cluster with coherently expressed gene members): MHC class I, type 1 IFN, and immunoglobulin signatures. The type 1 IFN, MHC class I, and immunoglobulin signatures have been reported previously in various subclasses of myositis, in good agreement with the results in this study. Also, the current study is the first to systematically evaluate these genomic signatures together, which could provide important insight into the pathogenesis and potential therapeutic targets for myositis. Several transcripts displaying a good correlation with the leukocyte index and/or the type 1 IFN signature in muscle were also identified in this study, such as TTN, miR-1, miR-155 and amyloid beta (A4) precursor protein-binding, member B and member 1 interacting protein (APBB1IP). However, the small sample size and insufficient clinical information preclude us from evaluating the association of these molecular characteristics with the disease activity/severity within this study. Additionally, there was not adequate material to conduct extensive immunohistochemistry studies on patients in this study to correlate the presence of immunoglobulin and MHC class I protein levels with the corresponding gene signature scores. Continued effort to evaluate these molecular features in clinical studies where detailed clinical/demographic information is available will allow for further characterization of the utility of these potential biomarkers to aid in the patient stratification or to predict therapeutic efficacy.

In contrast to the positive correlation identified between the type 1 IFN signature and leukocyte infiltration index in PM and IBM, a positive association was not observed in most DM samples. One possible explanation is that the type 1 IFN signature in PM and IBM is mainly driven by leukocyte infiltration into muscle, while distinct drivers might be present in DM. Our results support the previous finding [14] that the type 1 IFN signature in DM might be connected with PFA (Figure 4), although a larger sample size is desired. Notably, this association between PFA and the type 1 IFN signature was also accompanied by the presence of the MHC class I signature (see Figure 2B, C). Additional studies need to be carried out in the future to confirm these observations.

The ability to distinguish the role of different cell types at disease sites is important in identifying anti-correlations between miRNA and mRNA expression, such as the expression of miR-29c and collagen genes (Additional file 2: Supplementary Text and Supplementary Methods) and other anti-correlations that may be important to the pathogenesis of myositis. Anti-correlations comparing mRNA and miRNA expression levels could also be complicated by the effect of increased leukocyte cell abundance, potentially masking true correlations between mRNAs/miRNAs in various disease settings. The positive correlation identified between miR-146a and the type 1 IFN signature in PM and IBM is one example where the increased number of leukocytes in muscle influenced the resulting gene expression comparison between myositis patients and normal subjects. Taken together, these results strongly support the application of the leukocyte infiltration index to transcriptomic studies to appropriately characterize altered mRNA and/or miRNA expression from heterogeneous biological samples, thus allowing for more meaningful interpretation of microarray data with the aim of increasing our understanding of the fundamental mechanisms of disease pathogenesis.

Evaluation of the effect of miR-146a in the suppression of the type 1 IFN pathway in myositis muscle revealed an interaction that was confounded by leukocyte infiltration. Nevertheless, our results indicated that a subset of DM patients had high type 1 IFN signatures and low miR-146a expression in muscle. Additional *in vitro* results showed that altered miR-146a levels in muscle cells could regulate the activation of the type 1 IFN pathway. Due to the small number of myositis samples displaying an anti-correlation between miR-146a and the type 1 IFN pathway and the lack of *in vivo* studies confirming this relationship, the exact functional role of miR-146a in myositis remains uncertain. However, continued work on characterizing this relationship in myositis is warranted.

## Conclusions

In this study comparing transcriptional changes between myositis muscle biopsies and normal controls, we identified several gene signatures, including a leukocyte index and a type 1 IFN signature, as genomic biomarkers to characterize myositis subjects at the molecular level. Additionally, use of the leukocyte index to account for transcriptional changes due to increased lymphocyte infiltration into muscle revealed that the majority of these changes are driven by increased leukocyte abundance. Investigation into the relationship between leukocyte infiltration and a type 1 IFN signature suggested that increased ISGs might have two distinct sources at the myositis disease site: one is closely associated with leukocyte infiltration in IBM, PM and some DM; and the other source is not certain, but may be due to MHC class I-expressing myofibers, especially in some DM patients. Further understanding of the relationship between these signatures *in vivo*, as well as any association with myositis disease severity or activity will be explored in future clinical studies to evaluate their utilities as biomarkers. The ability to differentiate the transcriptional alterations due to an increased proportion of inflammatory cells from other changes at the expression level increases our ability to connect changes in specific cellular subsets with important biological phenomena critical for disease development or progression.

## Methods

### Patients and tissue samples

Muscle samples from 36 patients (5 normal controls, 5 NM, 8 DM, 8 PM and 10 IBM) were studied (Table 1). Normal controls are those subjects who were not suspected clinically to have neuromuscular disease; had normal muscle strength by examination; and showed normal serum CK levels. Diagnostic criteria for IBM, DM, and PM were as previously described [11]. Patients with NM had an acute or subacute myopathy responsive to immunotherapy, with pathology showing necrotic muscle fibers without inflammatory cells other than macrophages. Myositis-specific antibodies may also be highly predictive of the clinical pheno-subtype of myositis; however, these data were not available for the patients utilized in this study and could not be incorporated into the overall phenotyping of the samples.

Open muscle biopsies were performed at the time of a diagnostic biopsy (the biopsy site is listed in Table 1) and immediately frozen in liquid nitrogen for RNA and miRNA. A separate piece of muscle was used for hematoxylin and eosin (H&E) staining. Patients provided informed consent for research and institutional review boards approved all studies.

### Hematoxylin and eosin (H&E) staining and assessment

H&E stained frozen sections were examined microscopically by a single examiner blinded to diagnosis. The degree of inflammation graded between 0–4 (0=no inflammation, 1=sparse inflammation, 2=mild inflammation, 3=moderate inflammation, and 4=severe inflammation).

### Total RNA extraction

miRNA-RNA was extracted from muscle biopsies using the mirVana miRNA Isolation kit (Applied Biosystems/Ambion, Austin, TX), according to manufacturer’s instructions.

### mRNA profiling by Affymetrix microarray

Small species RNAs (e.g. miRNA, snRNA, tRNA) were removed from a volume of each miRNA-RNA sample using Agencourt RNAclean magnetic beads (Beckman-Coulter, Brea, CA). The resulting total RNA was profiled using Affymetrix Human Genome U133 plus 2.0 GeneChips® (Affymetrix, Santa Clara, CA). A selection of genes with high expression intensities identified by microarray were validated by quantitative Real-Time PCR (Additional file 2: Supplementary Methods; Additional file 2: Figure S4).

### miRNA profiling by ABI TaqMan Low-Density Array (TLDA)

miRNAs were profiled using TLDA microRNA Cards v1.0 (Applied Biosystems, Foster City, CA). Single-stranded cDNA synthesis from 100 ng of total miRNA-RNA and pre-amplification of specific miRNA targets was performed according to the manufacturer’s protocol. The microRNA cards were loaded and run on an Applied Biosystems 7900HT Real-Time PCR system.

### Normalization of microarray and TLDA expression data

Probe-level summaries of microarray expression data were calculated using fRMA, which was implemented in *R* Bioconductor (http://bioconductor.org). The microarray expression data and the normalized expression data matrix were deposited at the NCBI Gene Expression Omnibus (accession number GSE39454). For quantitative RT-PCR, data analysis of *Ct* values was conducted with SDS v2.2.2 software (Applied Biosystems). All samples were normalized to the mean *Ct* value of the endogenous control transcript RNU48.

### Statistical analysis

All statistical analyses were performed using *R* (http://www.r-project.org). Two group *t*-test was applied to identify differentially expressed mRNA/miRNAs in myositis muscle biopsies compared to the normal controls. The empirical Bayes method (from Bioconductor *limma* package) was also employed to identify myositis subclass-specific expression patterns [37]. In both of the overall and subclass-specific comparisons, the transcripts with average fold change > 4 and adjusted *p*-value for Benjamini-Hochberg < 0.05 are considered significant.

### Invasion model

Measurements of gene expression from heterogeneous samples are typically confounded by the abundance of the constituent cell types, which generally can be formulated as a linear model. In this study, we considered a special case of the linear model, where the proportion of a certain subset *t* of cell types increases significantly in a certain condition (e.g., the disease state) compared to a normal condition. We named this model the *invasion model*. On the basis of some simple interpolation (see Additional file 2: Supplementary Methods for details), the invasion model characterizes several valuable outcomes when the special case holds: 1) variations of the gene expression will result predominantly from the alterations in proportions of the cell type *t*; 2) the *t* cell type-specific gene will be co-overexpressed even if there is either no or negative correlation between gene expression; 3) the average of the fold changes of the *t* cell type-specific gene expression is a good indicator of the increase in the fraction of the cell type *t* in a state change, for example, from the normal state to a disease state. The portions of invading cell types (or subsets) may change in distinct patterns and thus multiple cell type-specific gene signatures will be identified in that case.

The invasion model can be readily applied to myositis to assess the leukocyte infiltration into the disease site. Abundance of leukocytes is low in normal skeletal muscles and may increase dramatically in inflammatory myopathies. Given the existence of some mRNAs and/or miRNAs predominantly expressed in leukocytes, we should observe strong concordance among the overexpression of those particular protein-coding and/or non-protein coding transcripts. In practice, constant expression of those transcripts is not a requisite in the invasion model as long as the variation of the expression is minor relative to the variation of the lymphocyte cell abundance due to the infiltration, according to the equation (5) in Additional file 2: Supplementary Methods. It is anticipated that the leukocyte-specific transcripts should display “coherent” overexpression compared to the normal muscle biopsies and form one or more large clusters. As a result, the overexpression of those mRNA/miRNAs could serve as a gene signature to quantify the level of the infiltration of one or multiple subtypes of dominant leukocytes in myositis muscle.

### Consensus clustering and gene signatures

A consensus clustering approach was employed to determine the proper cluster numbers and the robust members within each cluster by using *R clusterCons* package [40]. Multiple clustering algorithms (including agglomerative nesting clustering (agnes), divisive analysis clustering (diana), *k*-means, partitioning around medoids (pam), and hierarchical clustering (hclust)) were applied with a bootstrapping approach. The clustering results were further merged to identify the consensus clusters and their robust members. The membership robustness of each gene ranged from 0 to 1, defined as the average connectivity between a gene and all other members of the cluster [40]. Thus, a robust member, by definition, should have a high score (i.e., close to 1). We empirically used 0.6 as the cutoff value of membership robustness and took any cluster with at least five robust members to define the gene signature. For each cluster, a gene signature was defined as the median of the fold changes of the robust members relative to the normal controls.

To reduce the scale of the clustering problem and exclude uninformative genes, we applied the clustering only on a subset (i.e., ~200) of the differentially expressed genes that had the highest expression variance across subjects as recommended by the *clusterCons* developers [40].

### Functional analysis of differentially expressed mRNAs

The differentially expressed transcripts are annotated in multiple ways: Ingenuity Pathway Analysis Tool (IPA; Redwood City, CA), hypergeometric enrichment of gene ontology (GO) terms, KEGG pathways (using Bioconductor *GOstats* package) and the gene sets from MSigDB (Molecular Signatures Database, http://www.broadinstitute.org/gsea/msigdb/index.jsp). *IPA microRNA Target Filter* is also utilized to explore the likely interaction between differentially expressed mRNAs and miRNAs (see Additional file 2: Supplementary Methods; Additional file 8: Table S7).

### C2C12 cell maintenance and transfection

C2C12 cells were purchased from American Type Culture Collection (ATCC, Manassas, VA) and cultivated in recommended media at 37°C in a humidified atmosphere with 5% CO_2_. Artificial miR-146a mimics (Dharmacon) and miR-146a inhibitors (Dharmacon), as well as the appropriate scrambled controls, were transfected into C2C12 cells with PrimeFect siRNA (Lonza) according to manufacturer’s protocol. 24 hours after transfection, cells were stimulated with mouse IFN-alpha (PBL interferon source) for 4 hours before RNA was isolated and profiled for alterations in IFN-inducible genes.

## Competing interests

The work described in this article was supported by MedImmune, LLC. WZ, KS, BWH, CM, LG, KR, LR, BJ and YY are full-time employees of AstraZeneca/MedImmune. NS and SAG have served as consultants for AstraZeneca/MedImmune. There is no significant financial or other competing interest in the work.

## Authors’ contributions

YY, NS, and SAG designed research; AAA consented subjects and performed the biopsies; KS, CM, LG, SAG performed research; WZ, BWH analyzed data; and WZ, KS, BWH, KR, LR, DF, BJ, SAG, YY wrote the paper. All authors’ read and approved the final manuscript.

## Pre-publication history

The pre-publication history for this paper can be accessed here:

http://www.biomedcentral.com/1755-8794/5/53/prepub

## Supplementary Material

Additional file 1 **Table S1. **837 differentially expressed transcripts in myositis compared to normal controls. Click here for file

Additional file 2 **Supplementary material. **Supplementary information and figures for the main manuscript. Click here for file

Additional file 3 **Table S2. ** Numbers of the differentially expressed transcripts in the subclasses of myositis compared to normal controls. Click here for file

Additional file 4 **Table S3. **69 differentially expressed miRNAs in myositis compared to normal controls. Click here for file

Additional file 5 **Table S4. **List of the gene members of the clusters A-E. Click here for file

Additional file 6 **Table S5. **Overexpressed transcripts after adjustment with the leukocyte index. Click here for file

Additional file 7 **Table S6. **Subclass-specific overexpressed transcripts after adjustment with the leukocyte index. Click here for file

Additional file 8 **Table S7. **miRNA-target mRNA pair candidates. Click here for file

## References

[B1] Arahata K Engel AG Monoclonal antibody analysis of mononuclear cells in myopathies I: quantitation of subsets according to diagnosis and sites of accumulation and demonstration and counts of muscle fibers invaded by T cells Ann Neurol 1984 16 193 208 10.1002/ana.410160206 6383191

[B2] Greenberg SA Bradshaw EM Pinkus JL Pinkus GS Burleson T Due B Bregoli L O'Connor KC Amato AA Plasma cells in muscle in inclusion body myositis and polymyositis Neurology 2005 65 1782 1787 10.1212/01.wnl.0000187124.92826.20 16344523

[B3] Greenberg SA Pinkus GS Amato AA Pinkus JL Myeloid dendritic cells in inclusion-body myositis and polymyositis Muscle Nerve 2007 35 17 23 10.1002/mus.20649 16969836

[B4] Salajegheh M Pinkus JL Amato AA Morehouse C Jallal B Yao Y Greenberg SA Permissive environment for B-cell maturation in myositis muscle in the absence of B-cell follicles Muscle Nerve 2010 42 576 583 10.1002/mus.21739 20740627

[B5] Antiga E Kretz CC Klembt R Massi D Ruland V Stumpf C Baroni G Hartmann M Hartschuh W Volpi W Characterization of regulatory T cells in patients with dermatomyositis J Autoimmun 2010 35 342 350 10.1016/j.jaut.2010.07.006 20843660

[B6] Arahata K Engel AG Monoclonal antibody analysis of mononuclear cells in myopathies III: immunoelectron microscopy aspects of cell-mediated muscle fiber injury Ann Neurol 1986 19 112 125 10.1002/ana.410190203 3008636

[B7] Arahata K Engel AG Monoclonal antibody analysis of mononuclear cells in myopathies. V: identification and quantitation of T8+ cytotoxic and T8+ suppressor cells Ann Neurol 1988 23 493 499 10.1002/ana.410230511 2968776

[B8] Engel AG Arahata K Monoclonal antibody analysis of mononuclear cells in myopathies. II: phenotypes of autoinvasive cells in polymyositis and inclusion body myositis Ann Neurol 1984 16 209 215 10.1002/ana.410160207 6089646

[B9] Greenberg SA Pinkus JL Pinkus GS Burleson T Sanoudou D Tawil R Barohn RJ Saperstein DS Briemberg HR Ericsson M Interferon-alpha/beta-mediated innate immune mechanisms in dermatomyositis Ann Neurol 2005 57 664 678 10.1002/ana.20464 15852401

[B10] Dalakas MC Review: an update on inflammatory and autoimmune myopathies Neuropathol Appl Neurobiol 2011 37 226 242 10.1111/j.1365-2990.2010.01153.x 21155862

[B11] Greenberg SA Sanoudou D Haslett JN Kohane IS Kunkel LM Beggs AH Amato AA Molecular profiles of inflammatory myopathies Neurology 2002 59 1170 1182 10.1212/WNL.59.8.1170 12391344

[B12] Tian L Greenberg SA Kong SW Altschuler J Kohane IS Park PJ Discovering statistically significant pathways in expression profiling studies Proc Natl Acad Sci U S A 2005 102 13544 13549 10.1073/pnas.0506577102 16174746 PMC1200092

[B13] Greenberg SA A gene expression approach to study perturbed pathways in myositis Curr Opin Rheumatol 2007 19 536 541 10.1097/BOR.0b013e3282efe261 17917532

[B14] Salajegheh M Kong SW Pinkus JL Walsh RJ Liao A Nazareno R Amato AA Krastins B Morehouse C Higgs BW Interferon-stimulated gene 15 (ISG15) conjugates proteins in dermatomyositis muscle with perifascicular atrophy Ann Neurol 2010 67 53 63 10.1002/ana.21805 20186858 PMC2875060

[B15] Banchereau J Pascual V Type I interferon in systemic lupus erythematosus and other autoimmune diseases Immunity 2006 25 383 392 10.1016/j.immuni.2006.08.010 16979570

[B16] Higgs BW Liu Z White B Zhu W White WI Morehouse C Brohawn P Kiener PA Richman L Fiorentino D Patients with systemic lupus erythematosus, myositis, rheumatoid arthritis and scleroderma share activation of a common type I interferon pathway Ann Rheum Dis 2011 70 2029 2036 10.1136/ard.2011.150326 21803750

[B17] Obermoser G Pascual V The interferon-alpha signature of systemic lupus erythematosus Lupus 2010 19 1012 1019 10.1177/0961203310371161 20693194 PMC3658279

[B18] Pascual V Farkas L Banchereau J Systemic lupus erythematosus: all roads lead to type I interferons Curr Opin Immunol 2006 18 676 682 10.1016/j.coi.2006.09.014 17011763

[B19] Ronnblom L Pascual V The innate immune system in SLE: type I interferons and dendritic cells Lupus 2008 17 394 399 10.1177/0961203308090020 18490415 PMC3694565

[B20] Assassi S Mayes MD Arnett FC Gourh P Agarwal SK McNearney TA Chaussabel D Oommen N Fischbach M Shah KR Systemic sclerosis and lupus: points in an interferon-mediated continuum Arthritis Rheum 2010 62 589 598 10.1002/art.27224 20112391 PMC2879587

[B21] Conigliaro P Perricone C Benson RA Garside P Brewer JM Perricone R Valesini G The type I IFN system in rheumatoid arthritis Autoimmunity 2010 43 220 225 10.3109/08916930903510914 20166872

[B22] Yao Y Richman L Morehouse C RM Dl Higgs BW Boutrin A White B Coyle A Krueger J Kiener PA Type I interferon: potential therapeutic target for psoriasis? PLoS One 2008 3 e2737 10.1371/journal.pone.0002737 18648529 PMC2481274

[B23] Wong D Kea B Pesich R Higgs BW Zhu W Brown P Yao Y Fiorentino D Interferon and biologic signatures in dermatomyositis skin: specificity and heterogeneity across diseases PLoS One 2012 7 e29161 10.1371/journal.pone.0029161 22235269 PMC3250414

[B24] Greenberg SA Dermatomyositis and type 1 interferons Curr Rheumatol Rep 2010 12 198 203 10.1007/s11926-010-0101-6 20425524 PMC2929916

[B25] Greenberg SA Higgs BW Morehouse C Walsh RJ Won KS Brohawn P Zhu W Amato A Salajegheh M White B Relationship between disease activity and type 1 interferon- and other cytokine-inducible gene expression in blood in dermatomyositis and polymyositis Genes Immun 2012 13 207 213 10.1038/gene.2011.61 21881594

[B26] Walsh RJ Kong SW Yao Y Jallal B Kiener PA Pinkus JL Beggs AH Amato AA Greenberg SA Type I interferon-inducible gene expression in blood is present and reflects disease activity in dermatomyositis and polymyositis Arthritis Rheum 2007 56 3784 3792 10.1002/art.22928 17968926 PMC2443782

[B27] Greenberg SA Type 1 interferons and myositis Arthritis Res Ther 2010 12 Suppl 1 S4 10.1186/ar2885 20392291 PMC2991777

[B28] Sibley CR Wood MJ The miRNA pathway in neurological and skeletal muscle disease: implications for pathogenesis and therapy J Mol Med (Berl) 2011 89 1065 1077 10.1007/s00109-011-0781-z 21751030

[B29] Eisenberg I Alexander MS Kunkel LM miRNAS in normal and diseased skeletal muscle J Cell Mol Med 2009 13 2 11 19175696 10.1111/j.1582-4934.2008.00524.x PMC3072056

[B30] Wang H Sun H Guttridge DC microRNAs: novel components in a muscle gene regulatory network Cell Cycle 2009 8 1833 1837 10.4161/cc.8.12.8851 19448406

[B31] Chen JF Callis TE Wang DZ microRNAs and muscle disorders J Cell Sci 2009 122 13 20 10.1242/jcs.041723 19092056 PMC2714401

[B32] Pauley KM Cha S Chan EK MicroRNA in autoimmunity and autoimmune diseases J Autoimmun 2009 32 189 194 10.1016/j.jaut.2009.02.012 19303254 PMC2717629

[B33] Dai R Ahmed SA MicroRNA, a new paradigm for understanding immunoregulation, inflammation, and autoimmune diseases Transl Res 2011 157 163 179 10.1016/j.trsl.2011.01.007 21420027 PMC3072681

[B34] Furer V Greenberg JD Attur M Abramson SB Pillinger MH The role of microRNA in rheumatoid arthritis and other autoimmune diseases Clin Immunol 2010 136 1 15 10.1016/j.clim.2010.02.005 20223711

[B35] Luo X Tsai LM Shen N Yu D Evidence for microRNA-mediated regulation in rheumatic diseases Ann Rheum Dis 2010 69 Suppl 1 i30 i36 10.1136/ard.2009.117218 19995741

[B36] David M Interferons and microRNAs J Interferon Cytokine Res 2010 30 825 828 10.1089/jir.2010.0080 20939680

[B37] Taganov KD Boldin MP Chang KJ Baltimore D NF-kappaB-dependent induction of microRNA miR-146, an inhibitor targeted to signaling proteins of innate immune responses Proc Natl Acad Sci U S A 2006 103 12481 12486 10.1073/pnas.0605298103 16885212 PMC1567904

[B38] Tang Y Luo X Cui H Ni X Yuan M Guo Y Huang X Zhou H De VN Tak PP MicroRNA-146A contributes to abnormal activation of the type I interferon pathway in human lupus by targeting the key signaling proteins Arthritis Rheum 2009 60 1065 1075 10.1002/art.24436 19333922

[B39] Lim LP Lau NC Garrett-Engele P Grimson A Schelter JM Castle J Bartel DP Linsley PS Johnson JM Microarray analysis shows that some microRNAs downregulate large numbers of target mRNAs Nature 2005 433 769 773 10.1038/nature03315 15685193

[B40] Simpson TI Armstrong JD Jarman AP Merged consensus clustering to assess and improve class discovery with microarray data BMC Bioinforma 2010 11 590 10.1186/1471-2105-11-590 PMC3002369 21129181

