# Supplementary material for: Genomic signatures characterize leukocyte infiltration in myositis muscles
Source: BMC Med Genomics. 2012 Nov 21;5:53. doi: 10.1186/1755-8794-5-53 (PMC3541209; doi:10.1186/1755-8794-5-53)
Supplement: Additional file 2 — Supplementary material. Supplementary information and figures for the main manuscript. [file 1755-8794-5-53-S2.doc]

# *Supplementary Information*

Supplementary Text

## Comparison of differentially expressed mRNAs among subclasses of myositis

To further identify subclass-specific differentially expressed transcripts (DETs), we compared the molecular profile from each individual myositis subclass to normal samples. Consistent with the previous two-group comparison, only a small fraction of transcripts are down-regulated (Additional file 3: Table S2). As for over-expressed genes, only two are differentially expressed in the comparison between NM and normal, while IBM samples display the largest numbers of DETs. Further pair-wise intersections among the DETs of IBM, DM and PM indicated that most DETs are altered in IBM only. Of the 103 DETs in PM compared to the controls, there are only two DETs unique to PM, with all remaining DETs commonly shared with IBM (Additional file 2: Fig. S1). There are no DETs in common between DM and PM other than the 21 DETs present in all three of the myositis classes (Additional file 2: Fig. S1). In contrast, 80 and 21 DETs are shared by IBM-PM and IBM-DM, respectively. Immunoglobulin, myosin, and MHC class II proteins are enriched in the former and interferon-stimulated genes (ISGs) are present in the latter. These results are consistent with observations from previous studies [1-3].

## Identifying mRNA target candidates of miRNAs dysregulated in myositis

In addition to investigating leukocyte infiltration and altered mRNA signatures in myositis, we further examined the presence of anti-correlations between altered miRNAs and mRNAs that may reveal important interactions that impact disease development. The predominant mechanism of target modulation by miRNA is translational inhibition of target mRNAs [4]. Accordingly, we identified 38 miRNA-target mRNA pair candidates with strong anti-correlated expression (see Additional file 2: Supplementary Methods; Additional file 8: Table S7). Among these candidates, the pair of miR-29c and collagen is both highly predicted by the database TargetScan and experimentally confirmed by quantitative RT-PCR and luciferase assay [5]. In this study, the expression changes of collagen genes (COL1A1 and COL1A2) display significantly negative linear correlation with that of miR-29c (*Spearman* rank test; both *r* = -0.8, *p*<0.001). The down-regulation of miR-29c was found in muscle disorders [6] and was also linked to muscle fiber loss and fibrosis in Duchenne muscular dystrophy [7]. Furthermore, collagen has been implicated in a feedback loop that directly inhibits muscle regeneration and directs the system toward fibrogenesis [8,9]. Consequently, the interaction between miR-29c and collagen genes might play an important role in muscle degeneration and myositis development. There are an additional 34 predicted mRNA:miRNA interactions with significant anti-correlations that have yet to be verified experimentally. They may also play a biological role in myositis.

Supplementary Methods

## Quantitative Real-Time PCR (qRT-PCR) validation of gene expression

## RNA was reverse transcribed into cDNA using SuperScript® III First-Strand Synthesis SuperMix kit (Life Technologies, Carlsbad, CA) with random hexamer priming. Samples were prepared using the TaqMan Pre-Amp Master Mix Kit (Applied Biosystems, Foster City, CA) and analyzed on BioMark 48.48 Dynamic Array chips with the BioMark Real-Time PCR System (Fluidigm Corp, South San Francisco, CA). Results were analyzed using BioMark Analysis software and cycle threshold (Ct) values above 30 were excluded from calculations. Delta-delta Ct values (ΔΔCt) were calculated using the mean of 4 reference genes (GAPDH, TFRC, β2M, and 18S) and a calibrator sample.

## mRNA:miRNA tnteraction analysis

IPA microRNA Target Filter was employed to identify the miRNA target genes. Differentially expressed miRNAs and mRNAs were uploaded to IPA and pairs of mRNAs:miRNAs with anti-correlation of expression patterns were selected as candidates. The correlation of each pair of the mRNA-miRNA interaction candidates was calculated and a conservative cutoff value was employed to further filter the candidates (Spearman rank test; *r* < -0.7, *p* < 0.001).

## Invasion model

For gene *i*, subject *j* and cell type *k*, the overall expression level of the gene *i* in the subject *j* can be defined as the sum of the product of the fraction of the cell type *k*, represented by$c_{j,k}^{s}$, and $x_{i,j,k}^{s}$, respectively. Thus, the expression level of the gene *i* within the cell type *k* at a state *s*:

$y_{i,j}^{s}=\sum_{k} c_{j,k}^{s}\cdot x_{i,j,k}^{s}$ (1)

where$\sum_{k} c_{j,k}^{s}=1$for each subject *j* under the state *s*.

Assuming that there is a gene set *I*, which are only expressed in a subset of cell types *T*, i.e., $x_{i,j,k}^{n}\approx0$ for $k\notin T$under any state *s*. Thus,

$y_{i,j}^{s}=\sum_{k\in T} c_{j,k}^{s}\cdot x_{i,j,k}^{s}=c_{j,t}^{s}\cdot x_{i,j,t}^{s}$, where $c_{j,t}^{s}=\sum_{k\in T} c_{j,k}^{s}$ and (2)

We may consider the mixture of the subset of cell types *T* as one special cell type *t*,

and its fraction $c_{j,t}^{s}$ is the sum of all the cell types T and the expression level $x_{i,j,t}^{s}$ of the gene *i* in the virtual cell type *t* is thereby the weighted arithmetic mean of the gene expression within the subset cell types *T*.

We may assume that $c_{j,k}^{n}$is sparse under the normal state n and $c_{j,k}^{d}$ increases significantly in a different state d (e.g., disease state), i.e., $c_{j,k}^{d}/c_{j,k}^{n}>>1$ (3)

And the log ratio of the fold change of the over-expressed transcripts between the disease state and the normal state:

$f_{i,j}=\ln(y_{i,j}^{d}/y_{i,j}^{n})=\ln\frac{c_{j,t}^{d}\cdot x_{i,j,t}^{d}}{c_{j,t}^{n}\cdot x_{i,j,t}^{n}}=rc_{j,t}+rx_{i,j,t}$ (4)

where $rc_{j,t}=\ln\frac{c_{j,t}^{d}}{c_{j,t}^{n}}$ and $rx_{j,t}=\ln\frac{x_{i,j,t}^{d}}{x_{i,j,t}^{n}}$ .
Therefore, in the log-transformed scale, the observed fold change can be attributed to two factors according to our model: the change of the cell fraction $rc_{j,t}$and the actual change of the gene expression $rx_{i,j,t}$. We may reasonably assume that $rx_{i,j,t}$ is subject-independent, independent of the change of the cell fraction$rc_{j,t}$, and an independently and identically distributed random variable following a normal distribution N(*μ*, *σ^2^*). For a pair of genes *g1*, *g2* belonging to the gene set *I*, $corr(f_{g1,j},f_{g2,j})=corr(rc_{j,t}+rx_{g1,j,t},rc_{j,t}+rx_{g2,j,t})=\frac{Var(rc_{j,t})+\rho\cdot\sigma^{2}}{Var(rc_{j,t})+\sigma^{2}}$

where $-1\leq\rho=corr(rx_{g1,j,t},rx_{g2,j,t})\leq1$. Therefore,

$corr(f_{g1,j},f_{g2,j})\to1$ , if $\sigma^{2}<<Var(rc_{j,t})$ (5)

The condition (5) generally holds due to the assumption (3) and we may select tightly co-expressed genes to further reduce$\sigma^{2}$. The model suggests that co-overexpression (i.e., positive correlation) of certain cell-type specific transcripts can be largely attributed to the change of the cell fraction even if there is either no or negative correlation between gene expression, i.e., $corr(rx_{g1,t},rx_{g2,t})\leq0$. Furthermore,

$E(f_{i,j})=E(rc_{j,t})+\mu$ (6)

Therefore, the average of the fold changes of the gene expression acts as a good indicator of increase of the fraction of the cell *t* in the sample, for example, from the normal state *n* to the disease state *d*.

Noticeably, the equation (5) also holds for a pair of mRNAs and miRNAs as long as these mRNAs and miRNAs are both specifically expressed within the common subset type of cells which also increase dramatically under the abnormal condition.

# Supplementary References

1. Greenberg SA, Sanoudou D, Haslett JN, Kohane IS, Kunkel LM, Beggs AH, Amato AA: **Molecular profiles of inflammatory myopathies.** *Neurology* 2002, **59:**1170-1182.

2. Tian L, Greenberg SA, Kong SW, Altschuler J, Kohane IS, Park PJ: **Discovering statistically significant pathways in expression profiling studies.** *Proc Natl Acad Sci U S A* 2005, **102:**13544-13549.

3. Greenberg SA: **A gene expression approach to study perturbed pathways in myositis.** *Curr Opin Rheumatol* 2007, **19:**536-541.

4. Guo H, Ingolia NT, Weissman JS, Bartel DP: **Mammalian microRNAs predominantly act to decrease target mRNA levels.** *Nature* 2010, **466:**835-840.

5. Sengupta S, den Boon JA, Chen IH, Newton MA, Stanhope SA, Cheng YJ, Chen CJ, Hildesheim A, Sugden B, Ahlquist P: **MicroRNA 29c is down-regulated in nasopharyngeal carcinomas, up-regulating mRNAs encoding extracellular matrix proteins.** *Proc Natl Acad Sci U S A* 2008, **105:**5874-5878.

6. Eisenberg I, Eran A, Nishino I, Moggio M, Lamperti C, Amato AA, Lidov HG, Kang PB, North KN, Mitrani-Rosenbaum S et al.: **Distinctive patterns of microRNA expression in primary muscular disorders.** *Proc Natl Acad Sci U S A* 2007, **104:**17016-17021.

7. Greco S, De SM, Colussi C, Zaccagnini G, Fasanaro P, Pescatori M, Cardani R, Perbellini R, Isaia E, Sale P et al.: **Common micro-RNA signature in skeletal muscle damage and regeneration induced by Duchenne muscular dystrophy and acute ischemia.** *FASEB J* 2009, **23:**3335-3346.

8. Alexakis C, Partridge T, Bou-Gharios G: **Implication of the satellite cell in dystrophic muscle fibrosis: a self-perpetuating mechanism of collagen overproduction.** *Am J Physiol Cell Physiol* 2007, **293:**C661-C669.

9. Gosselin LE: **Skeletal Muscle Collagen: Age, Injury and Disease.** In *Sarcopenia - Age-realated muscle wasting and weakness*. Edited by Edited by Lynch GS. Netherlands: Springer; 2011:159-172.

10. Simpson TI, Armstrong JD, Jarman AP: **Merged consensus clustering to assess and improve class discovery with microarray data.** *BMC Bioinformatics* 2010, **11:**590.

Supplementary Figures

**Figure S1: Venn diagram of the DM, PM and IBM-specific differentially expressed transcripts.**

**A**

**B**

**C**

**D**

**E**

#

**Figure S2**: **Consensus clustering of 197 overexpressed genes**

1. AUC plot showing that *k*-means clustering outperformed other clustering algorithms and merged results.
2. ΔK plot suggesting 13 is the optimal cluster number (see ref. 10 for the details of ΔK plot).
3. The box plot of the membership robustness of each cluster. The membership robustness of each gene ranged from 0 to 1, defined as the average connectivity between a gene and all other members of the cluster [10]. The five clusters above the empirically cut-off value of 0.6 (highlighted by the blue dot line) are selected and labeled by the letters A-E (in blue color).

**Figure S3**: **Parallel gene profile plots of the five robust clusters.** Each gene member of the cluster is represented by a line in the plot, while robust and non-robust members are colored in blue and grey, respectively. These five parallel profile plots indicate that the robust members of each gene cluster share highly similar expression pattern across the different samples.

**Figure S4: Quantitative Real-Time PCR (qRT-PCR) validation of microarray gene expression.** The Pearson’s correlation coefficients (*r*) and p-values (*p*) are listed in parentheses.
